# Management of Symptomatic and Asymptomatic May-Thurner Syndrome: A Review of Two Cases

**DOI:** 10.7759/cureus.106423

**Published:** 2026-04-04

**Authors:** Luis A Cruz Vásquez, Manuela Restrepo Gomez, Oscar F Vargas, Luis F Rivera

**Affiliations:** 1 Department of Interventional Radiology, Hospital General de Medellín, Clínica Especializada EMMSA, Medellín, COL; 2 Department of Diagnostic Radiology, Universidad Pontificia Bolivariana-Cedimed, Medellín, COL; 3 Department of Interventional Radiology, Hospital Departamental de Villavicencio, Villavicencio, COL

**Keywords:** deep venous thrombosis, endovascular surgical repair, iliac vein, may-thurner syndrome, venous stent

## Abstract

May-Thurner syndrome is a condition in which there is obstruction of blood flow in the left iliac vein due to compression between the right iliac artery and the spine. We present two clinical cases of women with venous compressive May-Thurner syndrome who underwent endovascular management with the insertion of a dedicated venous stent. One patient presented symptoms such as intermittent claudication, pain, and limb edema without other complications. The second patient had extensive deep venous thrombosis of the iliocaval axis and left femoral vein, for which endovascular aspiration thrombectomy was performed. Both procedures were technically successful, and no complications occurred.

## Introduction

May-Thurner syndrome is the most common cause of non-thrombotic venous occlusion in adults and accounts for 1-5% of chronic venous disease [1,2]. This syndrome results from the extrinsic compression of the left iliac vein as it passes between the right common iliac artery anteriorly and the fifth lumbar vertebra posteriorly, representing an anatomic variant present in approximately 20-25% of the general population [3]. It is more frequent in females and in patients in the second or third decade of life [4]. Risk factors include a history of multiple pregnancies, the use of oral contraceptives, dehydration, and prolonged periods of immobilization that lead to sustained venous stasis of the lower limbs [4]. The pathophysiology involves several sequential mechanisms: prolonged mechanical compression of the iliac vein causes repetitive pulsatile trauma to the venous wall, inducing intimal hyperplasia and scar formation with the development of fibrotic spurs or that progressively narrow the venous lumen [4]. 

May-Thurner syndrome has a wide variability of presentation and intensity of its clinical manifestations, and up to 66% of the general population may exhibit some degree of asymptomatic iliocaval extrinsic compression [2]. Typical clinical manifestations include edema and pain of the left lower limb accompanied by varicose veins, skin discoloration, and venous ulcers [3]. It has also been strongly associated with deep venous thrombosis due to venous stasis and reverse flow at this level. The most severe scenarios related to the syndrome include acute pulmonary embolism and phlegmasia [3].

Multiple radiological imaging modalities have been implemented for diagnosis. Selective venography with digital subtraction is the gold standard, as it is diagnostic and therapeutic [5]. Venography shows the stenosis of the left common iliac vein and the measurement of transvenous pressure gradients across the stenosis (pressure gradient >2 mmHg) and permits the identification of tortuous pelvic collaterals [5]. 

Doppler ultrasound is a study with few adverse effects, no radiation, and wide availability. However, in some patients, evaluation of the iliac vein is challenging due to anatomy or technical factors, making Doppler ultrasound a less reliable diagnostic method [5]. Therefore, contrast-enhanced computed tomography (CECT) in the venous phase and magnetic resonance imaging (MRI) allow the assessment of the degree of compression and the presence of associated thrombosis [6]. Likewise, intravascular ultrasound (IVUS) has also been implemented; this technique uses endovascular ultrasound to provide improved visualization of venous walls and areas of stenosis [2]. Nevertheless, its availability is limited, and its use is more restricted to therapeutic procedures rather than diagnosis [2].

The therapeutic strategy of choice is endovascular treatment with the implantation of vein-dedicated stents. Technical success rates with venous stents in the management of external compressions such as May-Thurner syndrome reach up to 100% [1]. The objective is to enlarge the caliber of the venous lumen and restore normal venous flow toward the inferior vena cava [7]. In some cases, it is associated with venous thrombosis treated with mechanical thrombectomy which consists in aspirating and fragmenting the thrombus without the need to infuse thrombolytic agents, which carry a high risk of bleeding [7]. Using these aspiration devices allows for the effective removal of acute, subacute, and chronic thrombi and reduces hospital stay [7].

## Case presentation

Clinical case 1

A 37-year-old female patient with a history of four previous pregnancies and use of oral contraceptives (Microgynon) presented with a three-year history of left iliac fossa pain associated with pelvic varices previously treated with endovascular coil embolization of the left gonadal vein, varicose veins in the left lower limb, and intermittent limb edema.

**Figure 1:**
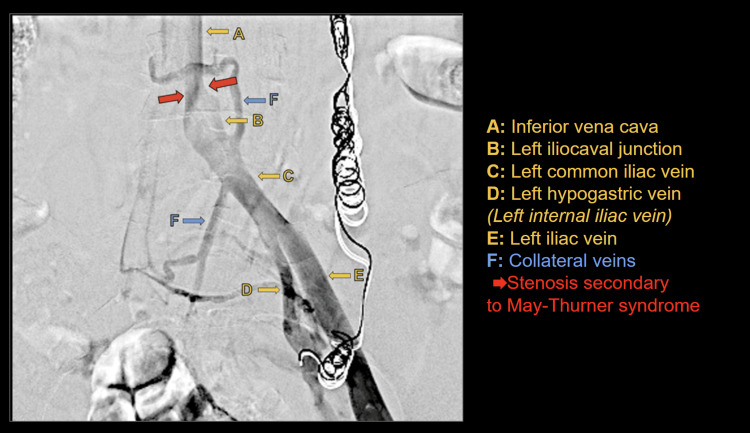
Near-occlusive stenosis of the left common iliac vein Patient with near-occlusive stenosis of the left common iliac vein at its junction with the inferior vena cava (red arrows and B). Patency of the inferior vena cava (A), left common iliac vein (C), left internal iliac vein (D), and left external iliac vein (E) is observed. Multiple collateral veins are also present (F). The patient has coils in the left gonadal vein from prior pelvic variceal embolization.

The procedure was performed under sterile conditions with local anesthesia infiltration in the left popliteal region. Under ultrasound guidance, puncture of the left popliteal vein was performed. Through the popliteal vein and advancing cranially, selective venography of the left lower limb was conducted, demonstrating adequate patency of the femoral vein, common femoral vein, and external iliac vein. The left common iliac vein, at its junction with the inferior vena cava, showed severe stenosis with synechiae formation and near-complete luminal occlusion (Figure 1). Endovascular repair was performed with the implantation of a vein-dedicated stent, Abre™ venous self-expanding stent system (Medtronic, Minneapolis, MN, USA), in the left iliac vein and the inferior vena cava.

**Figure 2:**
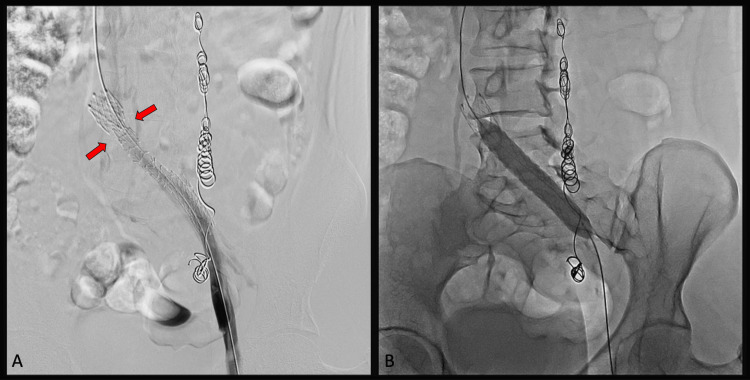
Insertion of a dedicated venous stent (A) Deployment of an Abre™ venous self-expanding stent system (Medtronic, Minneapolis, MN, USA) in the left common iliac vein at its junction with the inferior vena cava. The red arrows indicate the area of greatest compression, corresponding to the crossing of the right iliac artery. (B) Post-deployment stent dilation is performed using an Armada™ peripheral balloon (Abbott, Abbott Park, IL, USA) measuring 12 mm × 60 mm × 135 mm.

**Figure 3:**
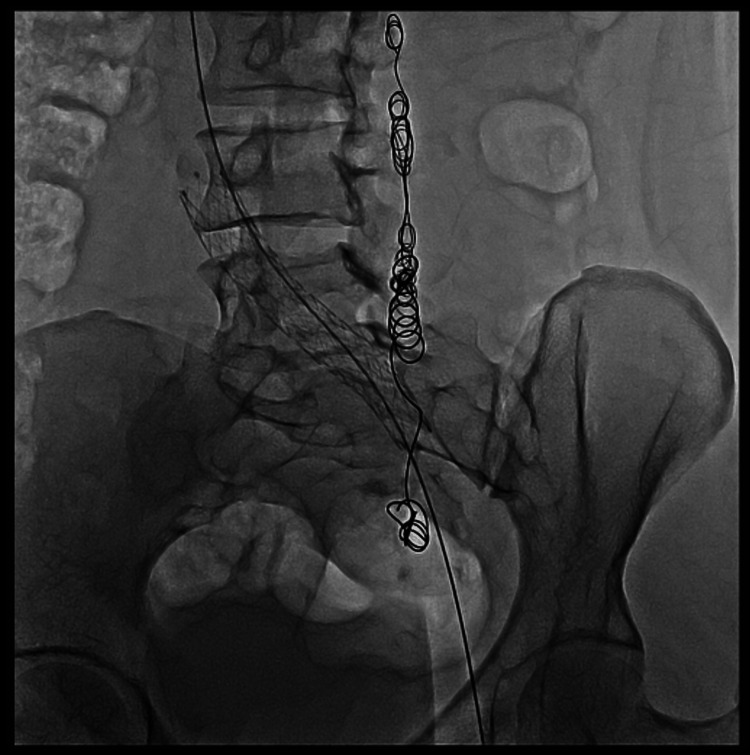
Venous stent functioning Proper positioning and functioning of the venous stent, with the normalization of the vascular caliber in a patient with May-Thurner syndrome.

Post-angioplasty and post-stent angiographic control of the left iliac vein and its junction with the inferior vena cava demonstrated the adequate restoration of normal vascular caliber and good antegrade passage of contrast toward the inferior vena cava. There was a complete disappearance of the collateral veins initially observed (Figure 2 and Figure 3). The patient tolerated the procedure well, with no complications.

Clinical case 2

A female patient presented with a long-standing history of left lower limb pain, accompanied by a sensation of heaviness and varicose veins on the left side. After diagnostic studies, an extensive iliocaval venous thrombosis of the left lower limb was identified, caused by venous stasis and the obstruction of venous outflow due to the extrinsic compression of the left iliac vein.

**Figure 4:**
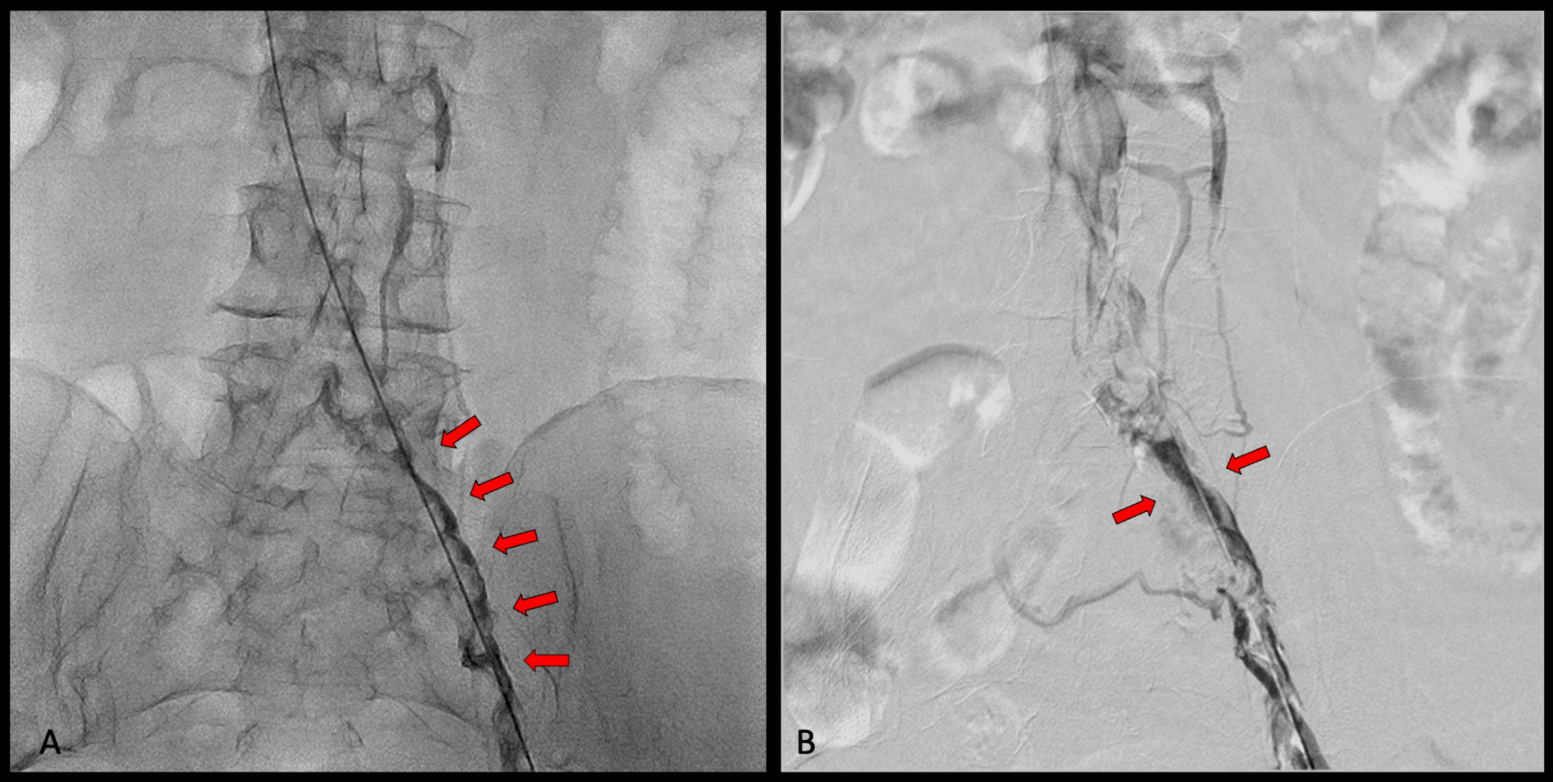
Acute deep venous thrombosis (A and B) Ascending selective venography of the left lower limb showing acute deep venous thrombosis throughout the entire popliteal and iliocaval axis. An area of obstruction is also seen at the junction of the inferior vena cava with the left common iliac vein.

**Figure 5:**
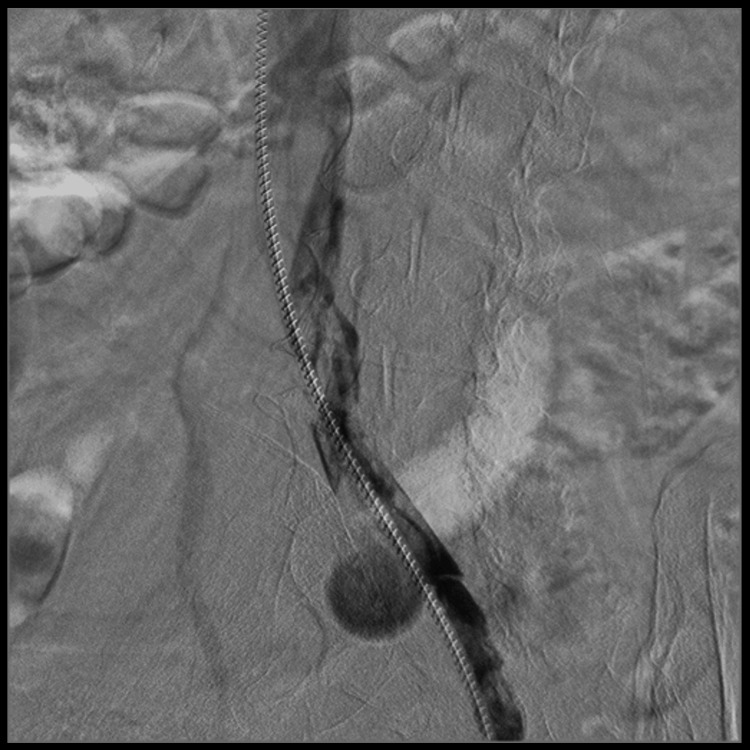
Aspiration of the thrombi Endovascular aspiration of the thrombi was performed using an Aspirex 10-French device. The Aspirex® thrombectomy catheter (Straub Medical, Wangs, Switzerland) is shown in the image.

Then an endovascular repair of May-Thurner syndrome associated with deep venous thrombosis of the left iliac vein was performed. The procedure was carried out with prior authorization and signed informed consent. Under sterile conditions, local anesthesia was infiltrated into the left popliteal fossa, and the left popliteal vein was punctured under Doppler ultrasound guidance using a micropuncture set. Through the popliteal vein and advancing cranially, selective venography of the left lower limb was performed, revealing acute thrombi throughout the femoropopliteal and iliocaval axis. The thrombosed segment was successfully crossed, and cavography of the inferior vena cava was performed, showing thrombi at its junction with the left common iliac vein (Figure 4). A 12-French vascular introducer was placed, and an Aspirex® thrombectomy catheter (Straub Medical, Wangs, Switzerland) was advanced to perform the endovascular thromboaspiration of the venous thrombi (Figure 5).

**Figure 6:**
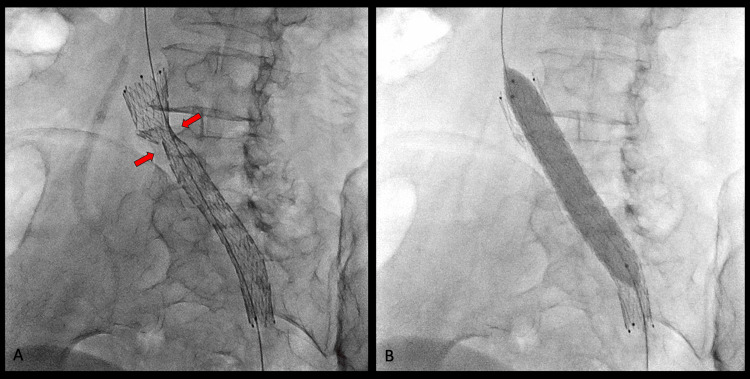
Deployment of a vein-dedicated stent (A) The stent is shown at the site of greatest obstruction of the left iliac vein, corresponding to the crossing of the right iliac artery, consistent with May-Thurner syndrome. (B) Balloon angioplasty was performed, successfully restoring normal vascular caliber.

**Figure 7:**
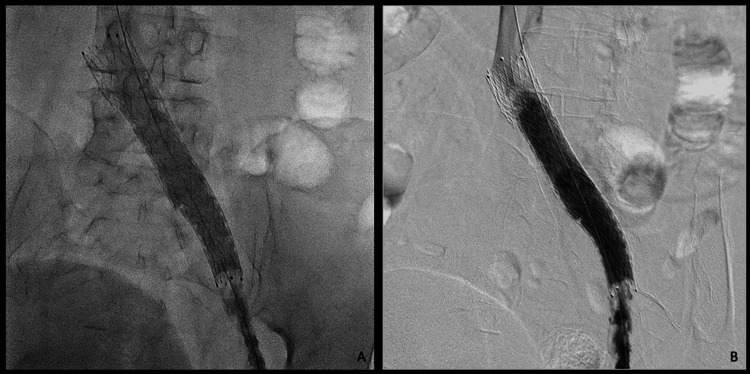
Restored vascular caliber (A and B) Left iliac vein with restored vascular caliber after thrombus aspiration, demonstrating good antegrade contrast flow toward the inferior vena cava.

Follow-up angiography demonstrated stenosis at the left iliocaval junction caused by extrinsic compression, consistent with May-Thurner syndrome. An Abre™ venous self-expanding stent system vein-dedicated stent was implanted, followed by balloon angioplasty with an Armada™ peripheral balloon (Abbott, Abbott Park, IL, USA) (Figure 6). Restoration of normal vascular caliber was achieved, with adequate antegrade passage of contrast toward the inferior vena cava (Figure 7). The patient tolerated the procedure well, without complications.

## Discussion

May-Thurner syndrome was first described in 1957 by May and Thurner [8], who observed that 22% of 430 cadavers presented a spur in the left iliac vein formed by chronic compression as it passed between the common iliac artery and the vertebral column [8]. It was not until 1965 that the term Cockett syndrome was introduced, when Cockett and Thomas described a series of 29 cases of iliofemoral thrombosis in which, using venography, they identified the extrinsic compression of the common iliac vein at the point where it is crossed by the right common iliac artery [9]. Currently, the condition is also referred to as non-thrombotic iliac vein compression syndrome, a more descriptive term that highlights the distinction between venous obstructions of thrombotic origin and those caused by extrinsic compression.

Since then, the number of reported cases in the literature has increased, underscoring the importance of appropriate diagnosis and management. In 2021, a meta-analysis was conducted on evaluating the treatment of venous disease with vein-dedicated stents, including 16 studies and more than 1,500 patients with deep venous thrombosis, among them non-thrombotic compressions such as May-Thurner syndrome [10]. The investigators found that dedicated venous stents demonstrate good safety profiles and lead to significant clinical improvement [10]. In a 2022 case series by Law et al., endovascular treatment with venous stents in patients with May-Thurner syndrome also had positive outcomes, with symptom improvement and maintained stent patency from six to 13 months [11].

Despite being an invasive technique that involves ionizing radiation exposure, venography remains the diagnostic study of choice for patients with pelvic venous disease, as it enables precise diagnosis and therapeutic intervention in the same session [1]. In both cases presented here, diagnosis was confirmed through an endovascular approach, with selective venography demonstrating significant stenosis, reduced caliber of the left iliac vein, and venous stasis. In both patients, the stenotic segment was located at the classic site of compression between the right iliac artery and the vertebral column.

The primary goal of treatment in May-Thurner syndrome is to restore venous flow, and two major therapeutic approaches exist: open surgery and minimally invasive endovascular therapy. Open surgery, historically known as the Palma procedure, described in 1960, consisted of a femorofemoral bypass [12]. Although initially considered a mainstay of treatment for iliac vein compression, it demonstrated high failure rates and numerous complications and is now largely obsolete. Minimally invasive endovascular therapy has become fundamental in managing compressive venous syndromes due to its reduced invasiveness, lower adverse event rates, and excellent clinical outcomes. This therapeutic modality involves the implantation of vein-dedicated stents, which reestablish luminal patency and restore venous flow. Technical success rates for venous stent placement in May-Thurner syndrome reach up to 100%, even when the inferior vena cava is involved [1]. When associated venous thrombosis is present, percutaneous endovascular aspiration thrombectomy must be performed during the same procedure. This technique uses an endovascular device with a rotating internal helix to aspirate and fragment thrombi. It allows for single-session management with a significantly lower bleeding risk compared to thrombolytic therapy [7].

Contemporary diagnostic algorithms increasingly advocate for IVUS as an adjunctive tool in the evaluation of iliac vein compression syndromes [13]. IVUS provides superior sensitivity for detecting non-thrombotic iliac vein lesions compared to conventional venography, with studies demonstrating that venography alone may underestimate the degree of stenosis by up to 30% [14]. Furthermore, IVUS facilitates optimal stent sizing and positioning, which are critical determinants of long-term patency [15]. Although IVUS was not available in our institution at the time of these procedures, its incorporation into clinical practice is strongly recommended when resources permit. Regarding long-term outcomes, contemporary series report primary patency rates of 79-100% at one year and 74-89% at five years following venous stent placement for May-Thurner syndrome [16,17]. Factors associated with improved patency include adequate stent diameter, complete coverage of the compressed segment, extension into the inferior vena cava when necessary, and appropriate post-procedural anticoagulation [2]. For patient selection, current guidelines suggest that intervention is most beneficial in symptomatic patients with >50% luminal compression and demonstrable hemodynamic impairment or clinical manifestations such as edema, venous claudication, or skin changes [18].

In both cases reported here, a single-step endovascular procedure allowed for accurate diagnosis and immediate therapeutic intervention. Diagnostic venography was first performed, followed by the implantation of a venous stent at the site of greatest obstruction of the left iliac vein. Both procedures were technically successful, with no complications. The second patient, who also had an extensive thrombus burden in the iliocaval axis, underwent aspiration thrombectomy during the same session without complications or bleeding. This single-session approach is particularly relevant in the Latin American context, where limited access to specialized vascular centers, economic constraints, and geographic barriers often prevent patients from returning for staged procedures.

## Conclusions

May-Thurner syndrome is caused by the non-thrombotic venous compression of the left iliac vein. However, it may occasionally be associated with deep venous thrombosis due to chronic stasis and reverse venous flow. Currently, diagnostic venography and the implantation of vein-dedicated stents play an important role in appropriate management. Vein-dedicated stents are self-expanding nitinol devices specifically designed for the venous system, featuring larger diameters, greater radial force to resist external compression, enhanced flexibility, and superior crush resistance compared to arterial stents. We presented two cases of women with May-Thurner syndrome who successfully underwent venous stent implantation, achieving the restoration of normal iliac venous caliber and flow. One patient also had extensive associated venous thrombosis, for which successful mechanical aspiration thrombectomy was performed during the same procedure. No complications occurred, in this small case series, suggesting that endovascular stent placement may be a viable treatment option for May-Thurner syndrome. Nonetheless, the small sample size limits generalizability, and larger prospective studies are needed to establish long-term outcomes.
